# Peripheral Neuropathy in Mitochondrial Trifunctional Protein Deficiency due to a Variant in *HADHA* Gene

**DOI:** 10.30699/ijp.2024.2010490.3163

**Published:** 2024-07-24

**Authors:** Samaneh Abedidoust, Reza-Shervin Badv, Amitis Saliani, Aileen Azari-Yam

**Affiliations:** 1 Department of Molecular Pathology and Cytogenetics, Children’s Medical Center, Tehran University of Medical Sciences, Tehran, Iran; 2 Department of Pediatric Neurology, Children’s Medical Center, Tehran University of Medical Sciences, Tehran, Iran; 3 William Harvey Research Institute, Queen Mary University of London, London, England

**Keywords:** HADHA gene, Fatty acid ß-oxidation, Mitochondrial trifunctional protein, Neuropathy, Whole exome sequencing

## Abstract

We report a 4.5-year-old girl with recurrent episodes of bilateral lower limb weakness following periods of upper respiratory tract infection since the age of 1.5 years. Nerve conduction velocity and electromyography studies suggested distal motor neuropathy. The whole exome sequencing analysis revealed a homozygous variant, c.955G>A (p.Gly319Ser), of the mitochondrial trifunctional protein α-subunit (HADHA) gene. This variant has already been reported as pathogenic in an Iranian consanguineous family with a probable diagnosis of Charcot-Marie-Tooth disease. In addition, this variant, in compound heterozygosity with another likely pathogenic variant, has been known to be linked with mitochondrial trifunctional protein deficiency.

## Introduction

Mitochondrial ß-oxidation of fatty acids (FAO) is the major source of energy for skeletal muscle, heart, and liver tissues. Fatty acid oxidation disorders are a group of autosomal recessive metabolic disorders characterized by disrupted fatty acid metabolism. 

The overall prevalence of fatty acid oxidation disorders is between 1 in 5,000 and 1 in 10,000 births. However, the prevalence varies greatly by disease type with medium-chain acyl-CoA dehydrogenase deficiency (MCADD) being the most prevalent (1). 

Fatty acids of total carbon atom number 12 and above are classified as long-chain fatty acids. Four enzymes are involved in the ß-oxidation of long-chain fatty acids: very long-chain acyl-CoA dehydrogenase (VLCAD), long-chain 2, 3-enoyl-CoA hydratase (LCEH), long-chain 3-hydroxyacyl-CoA dehydrogenase (LCHAD), and long-chain 3-ketoacyl-CoA thiolase (LCKT) (2).

Mitochondrial trifunctional protein (MTP) is a multienzyme complex that catalyzes three out of four steps in the oxidation of long-chain fatty acids. MTP is composed of four α-subunits (having LCEH and LCHAD activity and encoded by the *HADHA* gene), and four ß-subunits (having LCKT activity and encoded by the *HADHB* gene (2-5).

MTP deficiency is clinically heterogeneous including lethal neonatal cardiomyopathy and Reye-like syndrome as well as an infantile hepatic disease with hypoketotic hypoglycemia. The patients can also present with peripheral neuropathy, myopathy, or sudden death (6). Also, rhabdomyolysis can occur and lead to muscle pain and weakness (7, 8).

## Case Report

A 4.5-year-old girl, the second child of relative parents (first cousins) was born at 38 weeks of gestation. The pregnancy was uneventful. Her birth weight was 3400 g. Newborn screening for hypothyroidism, maple syrup urine disease, and glucose-6-phosphate dehydrogenase deficiency returned no abnormal results. No hypoglycemia was detected in the first hours after birth.

The patient’s developmental process including head holding, rolling over, walking, and talking was achieved within the normal time window. The older sibling was not affected, and the family history was negative for metabolic disorders.

At the age of 1.5 years, she was referred to the hospital with lower limb weakness and gait problems developed after 4 days of coryza symptoms and fever. Her cognitive state was intact. The blood pressure was 110/80 mmHg. Reduced muscle strength of the extremities (the proximal muscle strength: +2, the distal muscle strength: + 1) was detected. The lower extremity deep tendon reflexes were absent and dragging gait was noted. Clinical laboratory tests to rule out viral encephalitis including cerebrospinal fluid analysis and herpes simplex virus PCR (polymerase chain reaction) test were performed which returned negative results. The diagnosis of Guillain-Barré syndrome was made and the patient was treated with intravenous immunoglobulin (IVIG) therapy. One month later EMG and NCV studies were performed and showed axonal-type distal motor polyneuropathy.

At the age of 2.5 years, she was admitted to the hospital again for lower limb weakness and disability to stand following common cold, abdominal pain, and vomiting. Physical examination showed same results as the previous illness showed. Normal serum CK levels and normal urine analysis were repeatedly confirmed. Lactate levels during either episode of metabolic decompensation are not available. A mild increase in blood ammonia and homocysteine levels was identified. Plasma biochemical indexes are listed in Table 1. Pyruvate levels were not tested. Ophthalmic examinations were within normal limits. The electrocardiogram revealed normal sinus rhythm, mitral valve prolapses, and tricuspid regurgitation. Urine organic acid/acyl glycine and plasma organic acid/acylcarnitine profiles were measured using gas chromatography-mass spectrometry and liquid chromatography-tandem mass spectrometry. Blood acylcarnitine and urine organic acid analysis revealed normal levels. Analysis of plasma amino acids, and urine organic acid profiles revealed no abnormalities. The acylcarnitine profile examination was performed while the patient was metabolically stable and revealed no abnormality. Urine analysis was unremarkable.

Between the episodes of acute illness, the patient could walk independently but had a mildly limited ability to run, jump, and climb the stairs. She had no scoliosis or foot deformity. Nerve conduction velocity (NCV) and electromyography (EMG) studies were performed. The sensory nerve conductive velocities of both plantar and right median nerve were tested. Motor conductive velocities of the tibial, right peroneal, right median, and right ulnar nerves were tested. EMG was used to examine both anterior tibial muscles, right quadriceps femoris muscle, both biceps brachii muscles, both gluteus medius muscles, and left abductor pollicis brevis muscle. All sensory nerve action potentials (SNAP) and compound muscle action potentials (CMAP) were of low amplitude. Needle EMG revealed a neurogenic pattern. Thus, EMG and NCV studies suggested axonal-type distal motor polyneuropathy with myogenic changes in proximal muscles. The patient’s muscle biopsy and cultured skin fibroblasts were not available for study.

Genomic DNA analysis was performed on the genomic DNA extracted from the EDTA-anticoagulated peripheral blood (5 mL). Informed consent was obtained, and the ethical committee of Children’s Medical Center approved the study protocol. Whole exome sequencing (WES) was performed using the genomic Illumina Genome Analyzer platform (coverage: x200). 

A homozygous variant, NC_000002.12:g.26212590C>T, NC_000002.11:g.26435459C>T, NG_007121.2:g.37032G>A, NM_000182.5:c.955G>A, NM_000182.4:c.955G>A, NP_000173.2:p.Gly319Ser, ( rs752317877) was identified in the *HADHA* gene. This variant is of extremely low frequency in the genome Aggregation Database (gnomAD) and in silico prediction tools support a deleterious effect of this variant. Targeted Sanger sequencing confirmed the homozygous state of this variant in the proband as well as the carrier state of her parents and her sister.

## Discussion

Here, we report a patient who experienced muscular weakness during episodes of metabolic decompensation with onset at the age of 1.5 years. All metabolic screening studies including acylcarnitine profile, urine chromatography, and plasma amino acid high-performance liquid chromatography (HPLC) were performed when the patient was metabolically stable and did not disclose any abnormalities. The creatinine kinase levels were normal both during and between the decompensation episodes. Electrophysiologic studies suggested peripheral polyneuropathy with myogenic changes. Regarding the parents’ consanguinity and negative family history, an autosomal recessive pattern of inheritance was suggested. We performed WES which discovered a homozygous variant of uncertain significance (VUS) in the *HADHA* gene which encodes two out of three enzymes of the MTP. 

MTP is a complex of multiple enzymes for long-chain fatty acid β-oxidation. In MTP deficiency, patients may be asymptomatic when energy demand is low; however, in states such as infection, physical exercise, or prolonged intervals between meals, the symptoms appear as the metabolic system cannot provide for the high energy demand (1, 9).

In MTP deficiency, acylcarnitine analysis shows that the serum C16-OH, C16:1OH, C18-OH, and C18:1OH are increased and the urine organic acid profile demonstrates 3-OH-dicarboxylic aciduria. However, these analyses may not detect any abnormality when the patient is not metabolically in demand (10). Our patient had normal plasma amino acids, serum acylcarnitine profile, and urine organic acids with all the analyses performed between the episodes of metabolic decompensation.

MTP deficiency is a rare metabolic disorder that is classified into three groups according to the clinical manifestations: “1) lethal phenotype (neonatal-onset, severe form), 2) hepatic phenotype (infantile-onset, intermediate form), and 3) neuromyopathic phenotypes (late adolescent onset, mild form)” (11, 12). About 20 patients with neuromyopathic phenotypes have been reported so far in the western countries. Spiekerkoetter et al. reported 11 MTP-deficient patients with neuromyopathy with an age of onset range between 1 and 13 years (9). 

Up to date, 28 different mutations have been described in the *HADHA* gene of MTP-deficient patients (reviewed in the *HADHA* gene mutation database at the URL http://www.hgmd.org/). The first described and the most common pathogenic variant of LCHAD deficiency is a c.1528G>C mutation which substitutes glutamine for glutamic acid (E510Q) in the HADHA exon 15 (5, 13). In our patient a homozygous variant, c.955G>A, p.Gly319Ser in exon 10 of the *HADHA* gene (NM_000182.4) was detected. This variant has previously been reported in three patients. By NGS data analysis and searching specifically for the HADHA and HADHB disease-causing variants, Diebold et al. reported three patients in a 403-patient peripheral neuropathy/ myopathy cohort. Two patients (1 & 2) each had two heterozygous variants in HADHA. Patient 3 had a homozygous HADHB variant. Patient 2 had the c.453+1G>T (p.?) and c.955G>A (p.Gly319Ser) variants in the *HADHA* gene. They classified the first variant as likely pathogenic and the second one for which our patient is homozygous, as a variant of uncertain significance (VUS) (14). In a more recent study from Iran, Khani et al. presented two siblings with a clinical diagnosis of Charcot–Marie–Tooth (CMT) disease from a consanguineous family. The affected members presented with weakness in the lower extremities since early childhood. WES analysis revealed the same homozygous variant as our patient in the HADHA gene. The authors emphasized that the patients had a pure CMT2 presentation and suggested *HADHA* as an unusual disease‑causing gene (15). The HADHA:c.955G>A:p. The Gly319Ser variant is of extremely low frequency in gnomAD population databases. This variant is not in the functional domain of the gene; however, it replaces amino acid serine for glycine. These residues have different polarity, charge, hydrophobicity, and size. Glycine is a non-polar amino acid, while serine is polar with a neutral charge. In silico prediction, tools suggest a detrimental effect of this variant. To our knowledge, no functional study has been performed for this variation. According to the available data, the variant was interpreted as a variant of uncertain significance.

## Conclusion

In conclusion, we studied a patient with MTP deficiency presented with neuropathy. The symptoms aggravated following seasonal upper respiratory tract infections. WES showed a homozygous VUS in the HADHA gene. We hope that recognition of this variant will pave the way for a conceivable prenatal molecular diagnosis for at-risk family members and also, further delineation of the molecular and clinical spectrum of the disease.
